# Supplementary material for: HIV policy legacies, pandemic preparedness and policy effort to address COVID-19
Source: PLOS Glob Public Health. 2023 Jun 26;3(6):e0001767. doi: 10.1371/journal.pgph.0001767 (PMC10292714; doi:10.1371/journal.pgph.0001767)
Supplement: S1 Table — (DOCX) [file pgph.0001767.s001.docx]

**S1 Table: Top and Bottom 10 Countries COVID-19 & HIV Policy Response Scores**

|  | **HIV Policy Scores** |  | **COVID-19 Policy Scores**  **(at 548 days)** | |
| --- | --- | --- | --- | --- |
|  | **OVERALL SCORE** |  | **ALL MEASURES SCORE** | |
| **Rank** | **Country** | **Score** | **Country** | **Score** |
| Top 10 Scores | Netherlands | 86 | Argentina | 73 |
|  | South Africa | 85 | Honduras | 72 |
|  | Portugal | 82 | Peru | 72 |
|  | Norway | 82 | Cyprus | 71 |
|  | France | 81 | Gabon | 70 |
|  | Italy | 78 | Chile | 70 |
|  | Belgium | 77 | Panama | 69 |
|  | Finland | 77 | Colombia | 69 |
|  | Croatia | 77 | Rwanda | 68 |
|  | Rwanda | 76 | Israel | 68 |
| Bottom 10 Scores | Maldives | 31 | Belarus | 33 |
|  | Tonga | 31 | Somalia | 31 |
|  | Micronesia (Federated States of) | 31 | Niger | 30 |
|  | Equatorial Guinea | 30 | Kiribati | 30 |
|  | Kuwait | 30 | Solomon Islands | 27 |
|  | Yemen | 30 | Vanuatu | 24 |
|  | Djibouti | 29 | Yemen | 24 |
|  | Tuvalu | 22 | Tanzania | 18 |
|  | UAE | 15 | Burundi | 16 |
|  | Iraq | 15 | Nicaragua | 13 |
