# Supplementary material for: HIV policy legacies, pandemic preparedness and policy effort to address COVID-19
Source: PLOS Glob Public Health. 2023 Jun 26;3(6):e0001767. doi: 10.1371/journal.pgph.0001767 (PMC10292714; doi:10.1371/journal.pgph.0001767)
Supplement: S2 Table — (DOCX) [file pgph.0001767.s002.docx]

**S2 Table. Rank of Average Index Scores**
