# Supplementary material for: HIV policy legacies, pandemic preparedness and policy effort to address COVID-19
Source: PLOS Glob Public Health. 2023 Jun 26;3(6):e0001767. doi: 10.1371/journal.pgph.0001767 (PMC10292714; doi:10.1371/journal.pgph.0001767)
Supplement: S3 Table — (DOCX) [file pgph.0001767.s003.docx]

**S3 Table. Descriptive Statistics for Low- and Middle-Income Countries**

| **Variable** | **Obs.** | **Mean** | **Std. dev.** | **Min** | **Max** |
| --- | --- | --- | --- | --- | --- |
| **HIV Policy Index Scores** | | | | | |
| Overall | 104 | 55.27 | 11.00 | 15 | 85.00 |
| Testing and Prevention | 104 | 45.16 | 19.52 | 0 | 93.80 |
| Health Systems | 101 | 58.24 | 18.11 | 0 | 92.90 |
| Structural | 104 | 48.00 | 15.18 | 11.10 | 80 |
| Clinical Treatment | 103 | 72.85 | 17.94 | 28.60 | 100 |
| **COVID-19 Policy Index Scores: First 30 days from the first care reporting** | | | | | |
| Containment and Closure | 103 | 47.97 | 23.63 | 0 | 90.08 |
| Economic Support | 103 | 13.99 | 16.37 | 0 | 62.50 |
| Health Systems | 104 | 35.80 | 13.09 | 0 | 59.78 |
| **COVID-19 Policy Index Scores: First 365 days (1 year) from the first case reporting** | | | | | |
| Containment and Closure | 103 | 53.88 | 15.62 | 3.89 | 86.20 |
| Economic Support | 103 | 38.01 | 21.47 | 0 | 77.05 |
| Health Systems | 104 | 56.29 | 10.22 | 19.99 | 74.96 |
| **COVID-19 Policy Index Scores: First 730 days (2 years) from the first case reporting** | | | | | |
| Containment and Closure | 103 | 49.76 | 14.65 | 3.63 | 73.40 |
| Economic Support | 103 | 33.81 | 21.09 | 0 | 74.04 |
| Health Systems | 104 | 65.63 | 10.60 | 19.99 | 81.50 |
| **Covariates** | | | | | |
| UHC service coverage index | 104 | 57.67 | 14.14 | 27.33 | 82.97 |
| HIV Prevalence | 104 | 2.15 | 4.87 | 0 | 27.34 |
| GDP per capita (log) | 104 | 7.76 | 0.92 | 5.61 | 9.40 |
| Polity Score  (-10: strongly autocratic,  +10: strongly democratic) | 104 | 3.40 | 5.40 | -9 | 10 |
