# Supplementary material for: HIV policy legacies, pandemic preparedness and policy effort to address COVID-19
Source: PLOS Glob Public Health. 2023 Jun 26;3(6):e0001767. doi: 10.1371/journal.pgph.0001767 (PMC10292714; doi:10.1371/journal.pgph.0001767)
Supplement: Attachment — Submitted filename: Response Memo_PloS Global Health.docx [file pgph.0001767.s007.docx]

March 4, 2023

Re: PGPH-D-22-01546, HIV Policy Legacies, Pandemic Preparedness and Policy Effort to Address COVID-19

Dear Dr. Standley,

Thank you for giving us the opportunity to revise and resubmit our manuscript. We found the reviewers’ comments exceedingly useful in assisting us to clarify and improve certain points in the manuscript. Below we detail how we have responded to your comments and each reviewer’s comments. Our responses are outlined in red font.

We have especially focused on expounding on our justification and premise for examining how past HIV policy effort might influence present COVID-19 response efforts. We try to clarify that we are primarily interested in the earlier stages of the pandemic response as our motivation is premised on the idea that countries with more experience and effort combatting HIV will have been better prepared for the pandemic in terms of infectious disease infrastructure/capacity, respect for health and human rights and experience with prevention policy (e.g., testing/contact tracing). We have also expounded upon the ways that the two diseases might be different, rendering the experience from HIV less useful.

We hope that with these revisions, the reviewers and editors will deem the manuscript worthy of publication.

Sincerely,

The Authors

RESPONSE TO REVIEWERS

RESPONSES TO EDITOR/REVIEWER COMMENTS:

Journal Requirements:

In the online submission form, you indicated that your data will be submitted to a repository upon acceptance.  We strongly recommend all authors deposit their data before acceptance, as the process can be lengthy and hold up publication timelines. Please note that, though access restrictions are acceptable now, your entire data will need to be made freely accessible if your manuscript is accepted for publication. This policy applies to all data except where public deposition would breach compliance with the protocol approved by your research ethics board. If you are unable to adhere to our open data policy, please kindly revise your statement to explain your reasoning and we will seek the editor's input on an exemption. Please be assured that, once you have provided your new statement, the assessment of your exemption will not hold up the peer review process.

While we plan to deposit our manipulated dataset to an open access repository, please note that all the data is already currently publicly available. We only used publicly available data sources for our analysis.

Additional Editor Comments (if provided):

Thank you for the submission. The piece addresses an important topic, and provides useful data concerning potential linkages between past pandemic policies and responses to the COVID-19 pandemic. However, in its current form, the manuscript has some substantial shortcomings that would need to be addressed before further consideration. Notably, the premise of why HIV policies could provide useful information on COVID-19 response efforts, given all the differences between the diseases and current events, will be important to expand upon. Given the duration and dynamic nature of the COVID-19 pandemic, the challenge of how policies have changed over time, during different epidemiological phases (which in turn vary by country) should also be addressed directly in the manuscript - if not via updating of the methods or inclusion of new policies (i.e. into 2022) then at least as a limitation to the study. Perhaps the most extreme example of this will be the case of China, whose COVID-19 policies have changed substantially in just the past few weeks, and which could not have been anticipated by this manuscript, but yet presumably has important repercussions on the study's findings. Finally, the research has implications for how countries/authorities can better take on lessons learned, from a policy standpoint, from past epidemic experiences, for improved future pandemic preparedness and response; it would be helpful to expand upon these repercussions and opportunities for recommendations in the Discussion section.

Thank you. We now elaborate on points of juncture and disjuncture between the two diseases more fully in both the Introduction and the Discussion. We have also added some discussion about lessons for improved pandemic preparedness. See track changes.

Please also see the detailed feedback from both reviewers, provided below, in revising your manuscript for resubmission.

[Note: HTML markup is below. Please do not edit.]

Reviewers' comments:

Reviewer's Responses to Questions

**Comments to the Author**

5. Review Comments to the Author

Please use the space provided to explain your answers to the questions above. You may also include additional comments for the author, including concerns about dual publication, research ethics, or publication ethics. (Please upload your review as an attachment if it exceeds 20,000 characters)

**Reviewer #1**: This is a useful question to research. However, I found the article difficult to follow and the import of the findings are not entirely clear nor adequately explained. The authors should be clearer about what is in each data set and do more to clearly distinguish the Ec Supp data from Containment and Closure data. Throughout the piece it would be helpful to remind readers of the distinction.

Thank you for your comment. We recognize that with so many different sub-indices that we are looking at comparing the HIV Policy Response to the COVID-19 policy response that the results can be hard to follow. We have moved Table A1 & A2 from the Appendix to the main manuscript, which summarizes each Index and sub-index. We hope this clarifies.

I would more clearly acknowledge the differences between HIV and COVID; for example the fact that HIV often affects marginalized and stigmatized groups and is passed sexually makes it different from COVID in very important ways. More discussion is needed about the findings and the possible reasons for the findings.

Thank you for these comments. We acknowledge that this is largely an exploratory study. We now acknowledge in both the Introduction and Discussion section (Limitations) that while COVID and HIV are comparable in some respects, there are significant ways that they vary- i.e., particularly in terms of risk groups affected and transmission routes.

However, our central argument is that HIV has garnered the single greatest amount of global health investment of all recent pandemics and touched every country in the world. In this respect, HIV shares with COVID-19 that it has broadly been viewed as a threat to public health in both high-income and low- and middle-income country settings and captured public and policymaker attention for a sustained period of time.

**Reviewer #2**: This paper examines correlations between patterns in HIV policies and COVID-19 policies to determine whether countries with strong HIV policies were better prepared to handle the COVID-19 pandemic.

This is an important question that gets at some of the underlying reasons that drive how different countries addressed the COVID-19 pandemic and provides lessons for future pandemic preparedness. However, the I think this study should do more to examine some of the nuances of this research question and provide stronger conclusions and policy guidance as a result.

Defining the research questions

The study should do more to justify the research question and explain why we might expect there to be links between HIV policy for a steady-state epidemic and a novel public health emergency.

Is the type of public health capacity needed to manage HIV in the 2017-2020 (when it is endemic and largely managed via the primary health care system) similar to what is needed to manage COVID-19 in the first two years of the pandemic? For example, do they require the same type of health infrastructure or not? For example, COVID is more dependent on acute care capacity whereas HIV relies primarily on outpatient.

Identifying the specific areas of overlap between HIV and COVID policy would strengthen the hypotheses and provide a clearer foundation for interpreting the results.

Thank you for this comment. We see now that more elaboration is necessary to justify the comparison. We have added some additional justification on p. 3-5 of the Introduction. While we agree that acute care capacity is more important for COVID-19 than HIV, we believe that other aspects of pandemic preparedness can be gleaned from countries’ experience with HIV.

We also now highlight the extensive policy learning that occurred from infectious disease experts who had spent their careers on HIV and how those lessons were applied and translated to COVID-19.

It may also be that policies mature between the first two years of an epidemic and 30-plus years into one. How relevant would we expect long-term HIV policies to match emergency-state COVID-19 policies?

We agree, though see above. The basic argument is that countries with better existing capacity from their experience with HIV were better prepared to respond to the public health challenge of COVID-19 than those who were less prepared. We admit that this may not be the most deterministic policy, but we do believe that policy legacies can be influential for reasons we have outlined. We have added extensive additional elaboration on p. 3-6 on why we think that investments in HIV may have prepared countries for COVID-19 responses.

Comment: The hypotheses could use some more justification so the reader understands clearly why the connections were made. For example, 30 days seems like a short time for “nuanced” policy to develop – most countries were still in crisis mode 60 or more days into the pandemic.

We examine relationships at three time points (30 days, 365 days and 568 days) to examine the evolution of these relationships.

We do this in order to recognize that responses may evolve and become more evidence-based/experience-based over time. However, our premise is also that countries with more developed policy experience around HIV will have been better prepared to tackle this novel threat.

Our hypothesis 2A suggests that we expect the relationship between HIV policy effort and COVID-19 policy effort to strengthen with time.

Methodology

Comment: For the methodology, missing data was ignored. But I would be concerned that the lack of a policy in a particular area should be taken into consideration. No data on a policy likely means the policy didn’t exist and perhaps it should be coded as a zero rather than missing. I would want to see how that change in methodology changed the results.

*Response*: In fact, the Oxford index does code missing data as 0, so this should abate the reviewers’ concern- see their description of their methods here from the Supplementary materials of their Nature article on the index:

“*We produce two versions of each index. One with the raw calculated index values, plus we*

*produce a "display" version which will "smooth" over gaps in the last seven days, populating*

*each date with the last available "good" data point. We also make the conservative assumption that an absence of data corresponds to a subindex score (Ij,t) of zero.”*

Hale T, Angrist N, Goldszmidt R, Kira B, Petherick A, Phillips T, et al. A global panel database of pandemic policies (Oxford COVID-19 Government Response Tracker). Nat Hum Behav [Internet]. 2021;5(4):529–38. Available from: <https://doi.org/10.1038/s41562-021-01079-8>

It would be helpful to have more justification for the methodological choices made in the study. For example, I didn’t understand why “An extra half-point is subtracted from each ordinal score if the policy [was?] targeted rather than general and with a missing value contributing 0.”

*Response*: The Oxford COVID-19 Government Response Tracker is not our dataset. It was created by Hale et al. at Oxford. We are using their scoring approach. However, I will note that I agree with their approach. If a policy only applies to part of a country rather than the whole country, we should not code the whole country as comporting with that policy. For instance, in a federalist system where certain states or provinces may have locked down but not others, we don’t want to give the highest stringency score to the whole country. We have added a note clarifying this point in the Methods section.

If the study data is updated daily, then perhaps the study time frame could be extended into 2022. As COVID-19 policy matured and many countries attempted to enter a “steady state” on COVID-19, I’d expect to see a stronger correlation with HIV policies.

*Response*: While we understand this impulse, theoretically, we are more interested in how HIV policy effort contributed to pandemic preparedness. We expect more convergence in policy responses over time as countries have undertaking policy learning during the pandemic. We are more interested in how the experience of HIV policy effort may have better prepared countries earlier in the pandemic.

I have several recommendations related to how the main results are presented:

The scatterplots are very hard to read. Seeing the cloud of points does not convey very useful information. I recommend simplifying their design and showing only the trend curve(s). For correlations, a line of best fit and/or correlation coefficients are a much more informative format than scatterplots alone.

Response: While we understand the Reviewer’s recommendation, we respectfully disagree. We believe there is value in a descriptive study in being able to see what countries fall on the extremes and the scatter around the trendline. However, we have now put the scatterplots in the Supplementary materials rather than have them featured in the main paper for those interested in seeing more detailed relationships. We hope this abates the concern.

I don’t think Table 1 adds very much useful information. You can simply say that the top ten top and bottom only have one in common (Rwanda) and don’t need to show the list.

Thanks for this note, we have moved this to the Supplementary materials for those who may be interested in more detail.

You mention potential confounders in the discussion session, but I think you can do more to address that limitation in the main results section. In general, “cross-country” regressions are not seen as particularly informative since comparing the US, for example, to Zambia isn’t something that one would do otherwise. I think it would be useful to perhaps break down the analyses by continent/region and OECD/non-OECD. The patterns that relate HIV policy to COVID policy are likely different based on country income, severity of the ongoing HIV crisis and level of infrastructure that can be reallocated to a public health emergency on short notice. It would add interesting results to the paper.

Response: Thanks for noting this. We have now added some control variables to our models: UHC service coverage index (from WHO)^[1]^ to capture health system capacity; HIV Prevalence (IHME, % of HIV/AIDS among populations 15-49 years) to capture severity of the HIV crisis; GDP per capita to capture income differences. We present our coefficient plots controlling for these variables. We also stratify our coefficient plots by high-income and low & middle-income countries. Neither of these changes/additions fundamentally alters our main findings.

Discussion and conclusion

With some adjustments to the results section, I think it would provide a much richer basis for a more detailed discussion section. The current discussion and (very short) conclusion sections leave the reader wondering what the contribution of this paper is to our understanding of how HIV policies influence responses to new public health threats. I think more effort to tease out impactful results will provide a stronger foundation for discussion, and ideally would lead to some clear take-home points for the reader.
*Response*: See revisions to the Discussion Section generally. We have tried to tease these out more.

For example, the connection between having strong social support policies for HIV and strong economic support policies for COVID is not all that surprising. Countries that have support for social policies generally apply them in multiple realms. It would be interesting to know whether this correlation is stronger or weaker depending on the level of GDP, when it might be the case that resources are not easily available for COVID social policies.

We have now added models that disaggregate between HICs and LMICs. We find similar patterns in the relationship whether we include or exclude HICs. While we understand the Reviewer’s point, we do not think this finding is obvious. Understanding the importance of economic support policies for infectious disease responses is not a given and many countries failed on this both during HIV and COVID. Prominent public health actors in the US argued that it was not their domain to consider economic support policies as part of the response.

I wonder whether countries with a strong biomedical infrastructure assumed it would be able to handle fallout from the pandemic and therefore didn’t invest in social support. Generally strong biomedical infrastructure is associated with resource-rich environments. But perhaps they overestimated their ability to control COVID?

We now include models both with and without HICs and find quite similar patterns. We agree with the reviewers assessment though that countries with strong biomedical infrastructures may have assumed they could handle the pandemic better and therefore not invested in social support. We have also added some further discussion of the fact that certain LMICs have more biomedical infrastructure than many people believe. For instance, the presence of the Pasteur Institute in Senegal bas been featured as having served a critical role for much of West Africa in managing the pandemic by providing testing capacity and expertise in evaluating treatments and monitoring emerging strains. South Africa was economically penalized for being among the first to identify emerging variants due to its strong infectious disease surveillance infrastructure. We now discuss these examples in the Introduction.

Smaller points

The references to tables A1 and A2 are reversed
Response: Thanks, we have corrected this.
The paper would benefit from more focused paragraphs and a tighter outline. It felt like it rambled a little bit too much in some sections.

It may be helpful to reference the tuberculosis literature as well since the field has struggled to find a good balance between human rights and slowing disease transmission. It has a dark history.

Response: Thanks for noting this. We have added discussion of Tuberculosis in the Introduction- p. 4.

6. PLOS authors have the option to publish the peer review history of their article ([what does this mean?](https://journals.plos.org/globalpublichealth/s/editorial-and-peer-review-process#loc-peer-review-history)). If published, this will include your full peer review and any attached files.


**Do you want your identity to be public for this peer review?** If you choose “no”, your identity will remain anonymous but your review may still be made public.


For information about this choice, including consent withdrawal, please see our [Privacy Policy](https://www.plos.org/privacy-policy).

Reviewer #1: No

Reviewer #2: No

[NOTE: If reviewer comments were submitted as an attachment file, they will be attached to this email and accessible via the submission site. Please log into your account, locate the manuscript record, and check for the action link "View Attachments". If this link does not appear, there are no attachment files.]

While revising your submission, please upload your figure files to the Preflight Analysis and Conversion Engine (PACE) digital diagnostic tool, <https://pacev2.apexcovantage.com/>. PACE helps ensure that figures meet PLOS requirements. To use PACE, you must first register as a user. Registration is free. Then, login and navigate to the UPLOAD tab, where you will find detailed instructions on how to use the tool. If you encounter any issues or have any questions when using PACE, please email PLOS at [figures@plos.org](mailto:figures@plos.org). Please note that Supporting Information files do not need this step.

1. Achieve universal health coverage, including financial risk protection, access to quality essential health-care services and access to safe, effective, quality and affordable essential medicines and vaccines for all
