# Supplementary material for: HIV policy legacies, pandemic preparedness and policy effort to address COVID-19
Source: PLOS Glob Public Health. 2023 Jun 26;3(6):e0001767. doi: 10.1371/journal.pgph.0001767 (PMC10292714; doi:10.1371/journal.pgph.0001767)
Supplement: Attachment — Submitted filename: RESPONSE MEMO.docx [file pgph.0001767.s008.docx]

**RESPONSE MEMO**

Re: HIV Policy Legacies, Pandemic Preparedness and Policy Effort to Address COVID-19 (PGPH-D-22-01546R2)

Date: May 16, 2023

Dear Editors,

Thank you for noting that we neglected to include Tables 1 & 2 in our manuscript even though they are cited in the text on p. 11. I’m not sure how they got dropped. They have now been put back in. We hope the paper is now complete.

Kind regards,

The Authors

-------

PGPH-D-22-01546R2
HIV Policy Legacies, Pandemic Preparedness and Policy Effort to Address COVID-19
Dr. Ashley Fox

Dear Dr. Fox,

We've checked your submission and before we can proceed, we need you to address the following issues:

1. Please include copies of Tables 1 and 2 which you refer to in your text on page 11.

We've returned your manuscript to your account. Please resolve these issues and resubmit your manuscript within 30 days. If you need more time, please email the journal office at [globalpubhealth@plos.org](mailto:globalpubhealth@plos.org). We are happy to grant extensions of up to one month past this due date. If we do not hear from you within 30 days, we will withdraw your manuscript on your behalf.

Please log on to PLOS Editorial Manager at <https://www.editorialmanager.com/pgph/> to access your manuscript. You will find your manuscript in the 'Submissions Sent Back to Author' link under the New Submissions menu. Be sure to remove your previous manuscript file if you are uploading a new file in response to these requests. After you've made the changes requested above, please be sure to view and approve the revised PDF after rebuilding the PDF to complete the resubmission process.

We are requesting these changes to comply with the PLOS Global Public Health submission guidelines (<https://journals.plos.org/globalpublichealth/s/submission-guidelines>). Please note that we won't send your manuscript for review until you have resolved the above requests. 

Thank you for submitting your work to PLOS Global Public Health and supporting our mission of Open Science.

Kind regards,
Oliver Ian Tolentino
PLOS Global Public Health
